# Tetraploid and hexaploid wheat varieties reveal large differences in expression of alpha-gliadins from homoeologous Gli-2 loci

**DOI:** 10.1186/1471-2164-10-48

**Published:** 2009-01-26

**Authors:** Elma MJ Salentijn, Svetlana V Goryunova, Noor Bas, Ingrid M van der Meer, Hetty C van den Broeck, Thomas Bastien, Luud JWJ Gilissen, Marinus JM Smulders

**Affiliations:** 1 Plant Research International, Wageningen UR, P.O. Box 16, NL-6700 AA Wageningen, the Netherlands; 2 Vavilov Institute of General Genetics, Russian Academy of Sciences, Moscow, 119991, Russia; 3 CGN, P.O. Box 16, NL-6700 AA Wageningen, The Netherlands; 4 Allergy Consortium Wageningen, P.O. Box 16, NL-6700 AA Wageningen, the Netherlands

## Abstract

**Background:**

Α-gliadins form a multigene protein family encoded by multiple α-gliadin (*Gli-2*) genes at three genomic loci, *Gli-A2*, *Gli-B2 *and *Gli-D2*, respectively located on the homoeologous wheat chromosomes 6AS, 6BS, and 6DS. These proteins contain a number of important celiac disease (CD)-immunogenic domains. The α-gliadins expressed from the *Gli-B2 *locus harbour fewer conserved CD-epitopes than those from *Gli-A2*, whereas the *Gli-D2 *gliadins have the highest CD-immunogenic potential. In order to detect differences in the highly CD-immunogenic α-gliadin fraction we determined the relative expression level from the homoeologous *Gli-2 *loci in various tetraploid and hexaploid wheat genotypes by using a quantitative pyrosequencing method and by analyzing expressed sequence tag (EST) sequences.

**Results:**

We detected large differences in relative expression levels of α-gliadin genes from the three homoeologous loci among wheat genotypes, both as relative numbers of expressed sequence tag (EST) sequences from specific varieties and when using a quantitative pyrosequencing assay specific for *Gli-A2 *genes. The relative *Gli-A2 *expression level in a tetraploid durum wheat cultivar ('Probstdorfer Pandur') was 41%. In genotypes derived from landraces, the *Gli-A2 *frequency varied between 12% and 58%. In some advanced hexaploid bread wheat cultivars the genes from locus *Gli-B2 *were hardly expressed (e.g., less than 5% in 'Lavett') but in others they made up more than 40% (e.g., in 'Baldus').

**Conclusion:**

Here, we have shown that large differences exist in relative expression levels of α-gliadins from the homoeologous *Gli-2 *loci among wheat genotypes. Since the homoelogous genes differ in the amount of conserved CD-epitopes, screening for differential expression from the homoeologous *Gli-2 *loci can be employed for the pre-selection of wheat varieties in the search for varieties with very low CD-immunogenic potential. Pyrosequencing is a method that can be employed for such a 'gene family-specific quantitative transcriptome profiling'.

## Background

Wheat (*Triticum *spp.) is one of world's major food crops. Products derived from wheat flour are consumed by humans in many forms such as bread, pasta and other baked foods, and wheat gluten are used as additives in sauces, soups and sweets. *T. aestivum *(2n = 6x = 42; AABBDD genome) is an allohexaploid that was formed through hybridization and successive chromosome doubling of three diploid *Triticum*/*Aegilops *species. The diploid ancestors of the D genome and the A genome of *T. aestivum *are respectively *A. squarrosa *(DD) and *T. urartu *(AA). *A. speltoides *(SS) species have been suggested as the ancestor of the B genome, but the exact diploid progenitor remains uncertain [1,2]. While *T. aestivum *varieties are used for bread making, tetraploid *T. turgidum *var. durum (AABB genome) varieties are especially suitable for pasta production. The composition of the gluten fraction of the wheat grain is essential for the industrial quality. The gliadins (α, β, γ and ω gliadins) and glutenins (HMW and LMW glutenins) are typical gluten components of *Triticeae *cereals and these protein types determine respectively the viscosity and elasticity (strength) of the dough [3,4].

Gluten proteins are also the cause of celiac disease (CD), a T-cell-mediated disease with prevalence between 0.5–2 percent in human populations [5]. The disease is characterized by a chronic intestinal inflammation upon ingestion of gluten peptides [6-8]. Gluten peptides derived from α-gliadins are especially immunodominant, inducing strong T-cell responses in the large majority of CD-patients, as compared to peptides derived from other gluten proteins such as γ-gliadins and glutenins [9]. Furthermore, α-gliadins harbour the p31-43/49 peptide that is assumed to trigger the innate response pathway [10,11]. The α-gliadins are encoded by the *Gli-2 *locus located on the short arms of three homoeologous wheat chromosomes in hexaploid wheat (6AS, 6BS and 6DS). The individual loci are designated as *Gli-A2*, *Gli-B2 *and *Gli-D2 *[12]. Each *Gli-2 *locus is complex as it contains clusters of α-gliadin gene copies, with different levels of gene dispersion [13]. Estimates for α-gliadin gene copy number range from 25–35 to perhaps even 150 copies per haploid genome [14-16]. Most of the copies contain in-frame stop codons [17], but as these are not frequently observed in expressed α-gliadin sequences, they probably are pseudogenes that are not expressed [18].

Variation in immunogenicity exists among cultivars [19,20] and work is in progress to identify wheat varieties with a reduced amount of CD-epitopes to be used in a breeding program. However, the development of molecular markers specific for presence or absence of CD-epitopes in these cultivars is hampered by the complexity of the loci that harbour gluten genes. Sequencing and phylogenetic analysis of genomic α-gliadin clones from hexaploid *Triticum *species and diploids representing the three different genomes showed that they could be distinguished according to their genome of origin (A, B or D) based on sequence homology as well as on a different average length of the polyglutamine repeat motifs [17]. These differences between genomic origins were also observed among genomic and EST sequences of α-gliadins from hexaploid bread wheat [17,21]. It was also observed that the frequency of the HLA-DQ8+ restricted T-cell epitope Glia-α and the HLA-DQ2+ restricted T-cell epitopes Glia-α2, Glia-α9, and Glia-α20 differed between α-gliadins from the three homoeologous genomes [17,20].

Analysis of α-gliadin transcripts (ESTs) showed that α-gliadins were preferentially expressed from the *Gli-D2 *locus whereas expression levels of α-gliadin genes from *Gli-B2 *were relatively low. Two peaks of expression were visible during seed maturaton: early (at 10 days post anthesis) and late (at 20 days post anthesis). Several α-gliadin genes from the *Gli-A2 *locus were expressed preferentially late in seed development [21].

In the present studies the composition of the *Gli-2 *transcriptome of wheat genotypes was analyzed in detail, showing the differential expression of *Gli-A2*, *Gli-B2 *and *Gli-D2 *α-gliadins among wheat cultivars. Genetic differences in the 5' part of the α-gliadin gene that are related to differences in CD-immunogenicity, were analyzed in detail. With the aim to develop a T-cell independent method to detect differences in the highly CD-immunogenic *Gli-2 *fraction of individual genotypes of wheat varieties, pyrosequencing was applied to quantify the frequency of *Gli-A2 *genes in the overall *Gli-2 *transcriptome. This assay will enable the pre-selection of wheat genotypes that are expected to differ in the amount of α-gliadin CD-epitopes in their gluten.

## Results

### Genetic composition of the Gli-2 transcriptome of selected wheat lines

**Figure 1:**
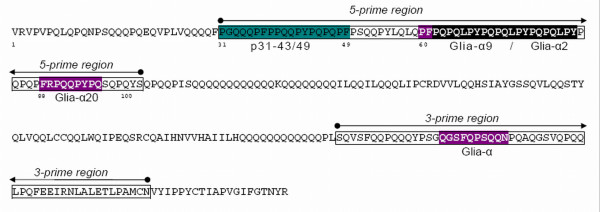
**Α-gliadin sequence with the position of CD-epitopes**. The sequence of a mature α-gliadin protein (gi|7209265|emb|CAB76964.1, *Triticum aestivum*) with the location of the respective epitopes for celiac disease depicted. p31-49, PGQQQPF*PP*QQ*P*YPQPQPF, triggering the innate immune response, the HLA-DQ2+ restricted T-cell epitopes Glia-α9(αI)(PFPQPQLPY), Glia-α9(αIII), (PYPQPQLPY), Glia-α2(αII) (PQPQLPYPQ) and Glia-α20 (FRPQQPYPQ) and the HLA-DQ8+ restricted T-cell epitope Glia-α (QGSFQPSQQN). Underlined Q is deamidated in the intestine by tTG.

The 5' part of the α-gliadin (*Gli-2*) gene is harbouring sequences coding for important HLA-DQ2+ CD-epitopes (Figure 1). With the aim to perform an initial assessment of genetic variation in transcribed *Gli-2 *genes and accompanying differences in the CD epitope frequency, *Gli-2 *transcripts from an individual plant of two hexaploid (*T. aestivum*) cultivars, 'Lavett' and 'Baldus', were analyzed in detail for their genomic origin (*Gli-A2*, *Gli-B2 *or *Gli-D2*) and for the presence of sequences encoding the CD-epitopes in the 5' part of the gene. The two cultivars are commercially grown in Europe [22]. 'Baldus' has its origin in The Netherlands, while 'Lavett' is from Sweden. They contain different HMW glutenin (*Glu-1*) subunits, which results in different scores for baking quality of 6 and 9, respectively [22]. In the assessment of the variation in *Gli-2 *transcripts we also included single genotypes of two landraces, 'Tripshiro' (CGN12287) and 'Sinde' (CGN12041) from the collection of the Centre of Genetic Resources, The Netherlands. 'Tripshiro' was collected as a tetraploid durum wheat in Libya and carries the HMW glutenin subunits *Glu-A1*c and *Glu-B1*b [23] whereas 'Sinde' originates from Ethiopia and was initially identified as hexaploid bread wheat (Table 1). Landraces are known to be more diverse and were included as a source for α-gliadin sequence variation and as a source of variation in expression among homoeologous loci. As landraces are often a mixture of genotypes, which are maintained by the genebank as such in order not to loose variation, we used seeds obtained from individual plants of these accessions.

**Table 1:** Wheat accessions

**Accession**	**Name**	***Triticum *spp**.	**Population type**	**Origin**	**Other information**
CGN23549	Lavett	*aestivum*	advanced cultivar	Sweden	Collecting date: 1997 [WW118466/Kadett//Dragon]
CGN19285	Baldus	*aestivum*	advanced cultivar	Netherl.	Collecting date: 1990 [Sicco/4/Sicco//N66/MGH653/3/Kolibri]
CGN0819	Deves	*durum*	landrace	Greece	Collecting date: 1916 (other name: Kamboyra)
CGN08403	x	*aestivum*	-	-	Collecting date: 1965 received as: *T. turgidum *group durum spring
CGN09696	x	*aestivum*	landrace	Pakistan	Collecting date: 1981 Farm store, altitude 2020m.
CGN10567	x	*aestivum*	breeding material	China	Collecting date: 1965 Selection from an Egyptian Landrace
CGN07991	St 472 Ethiopia	*durum*	landrace	Ethiopia	Collecting date: 1972; Local market, altitude 1830m. mixed with T. *turgidum *group durum
CGN16072	Dakar 52	*durum*	breeders variety	Egypt	Collecting date: 1965 Selection from an Egyptian Landrace
CGN08006	Dibillik Sinde	*aestivum*	landrace	Ethiopia	Collecting date: 1972; Local market, altitude 1830m. mixed with *T. turgidum *group durum
CGN08360	Diha Dzhavakhetskaja	*carthlicum*	landrace	Georgia	Collecting date: 1959
CGN16061	Tunisi	*durum*	unknown	Italy	Introduced from N.-Africa
CGN06575	Gandumi Saman	*aestivum*	landrace	Iran	Collecting date: 1940 received as: *Triticum durum *spring
CGN08262	Probstdorfer Pandur	*durum*	advanced cultivar	Austria	Collecting date: 1972 [Br 180/Wells]
CGN12287	Tripshiro	*durum*	landrace	Libya	Collecting date: 1921
CGN06582	Gaurani	*durum*	landrace	India	Collecting date: 1949
CGN12041	Sinde	*aesivum*	landrace	Ethiopia	Collecting date: 1972 Local market, altitude 1415m.

Passport details (CGN, Wageningen) of wheat accessions that were analysed by pyrosequencing for *Gli-A2 *allele frequency; 'landraces' are often a mixture of *T. aestivum *and *T. turgidum *genotypes. In brackets [], pedigree information. Abbreviations: *aestivum = T. aestivum; durum = T. turgidum *group durum; *carthlicum*. = *T. turgidum *group carthlicum; Netherl. = The Netherlands.

**Figure 2:**
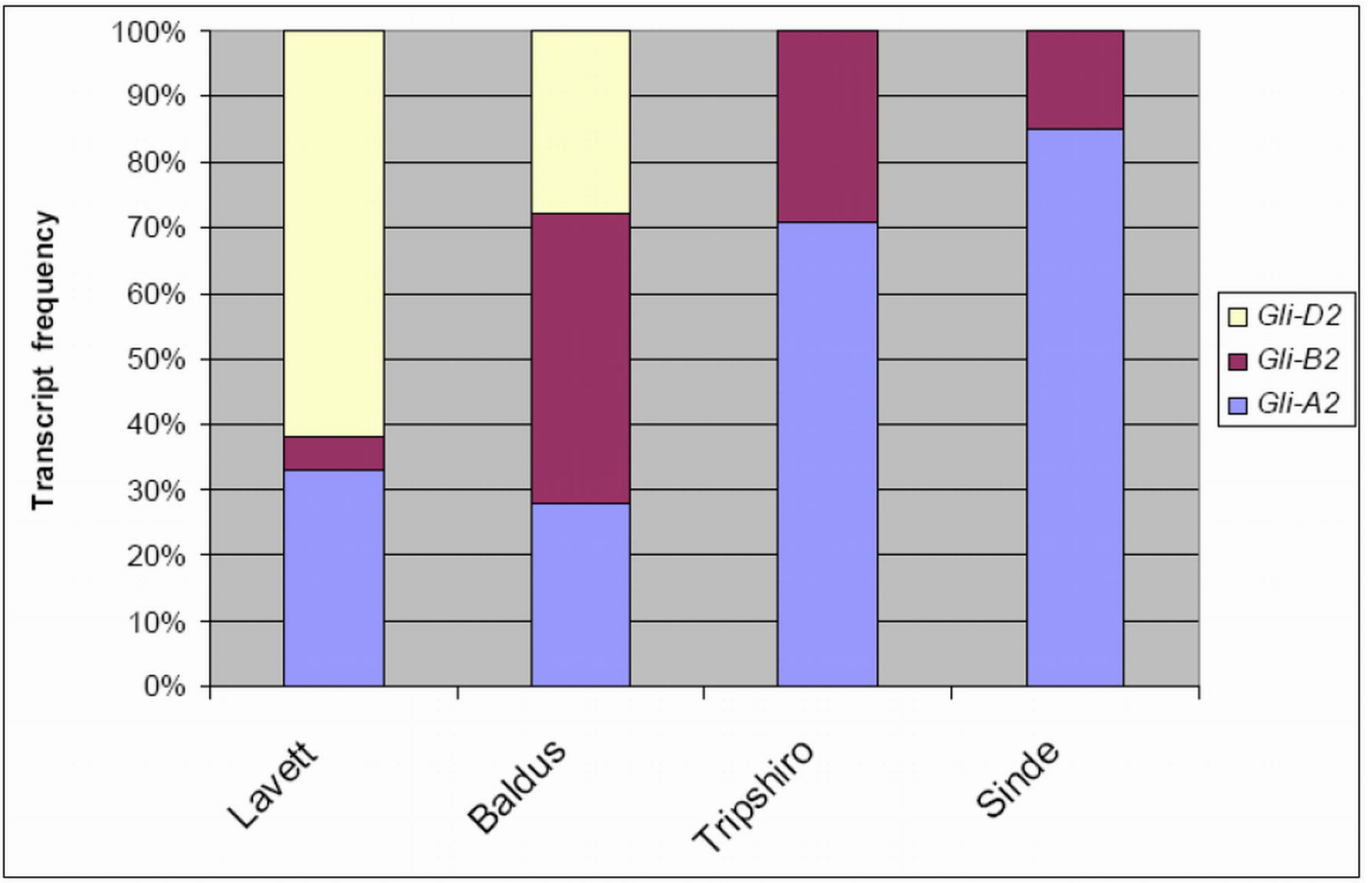
***Gli-A2*, *Gli-B2 *and *Gli-D2 *transcript frequencies in wheat genotypes**. Distribution of *Gli-A2*, *Gli-B2 *and *Gli-D2 *transcript frequencies; at 14 days post anthesis (DPA) as detected by cDNA sequencing in the transcriptome of two hexaploid bread wheat cultivars CGN23549 'Lavett' and CGN19285 'Baldus' and two genotypes derived from the landraces CGN12287 'Tripshiro' and CGN12041 'Sinde' respectively.

Α-gliadin sequences amplified from cDNA of the four different wheat lines were cloned and sequenced and subsequently assigned to one of the homoeologous *Gli-2 *loci (*Gli-A2*, *Gli-B2 *or *Gli-D2*) based on sequence homology as was done for genomic clones in Van Herpen et al. and Ma et al. [17,24]. Differential homoeoallelic expression patterns were observed for both the cultivars and the genotypes derived from the landraces. In the two hexaploid cultivars the *Gli-A2 *frequency was similar (33% for 'Lavett' and 28% for 'Baldus') whereas a large difference was observed in the expression rate of *Gli-D2 *and *Gli-B2 *genes; the ratio of expression *Gli-D2 *to *Gli-B2 *was 12.4 (62% *Gli-D2 *to 5% *Gli-B2*) for 'Lavett' but only 0.64 (28% *Gli-D2 *to 44% *Gli-B2*) for 'Baldus' (Figure 2). In the landraces we determined 71% *Gli-A2 *transcripts in the total *Gli-2 *transcriptome of 'Tripshiro' and 85% in 'Sinde' (Figure 2). In both cases this was the result of a low level of *Gli-B2 *sequences. Although 'Sinde' was listed as hexaploid *T. aestivum *(Table 1), the individual genotype analyzed here probably was tetraploid because there were no *Gli-D2 *sequences expressed.

**Figure 3:**
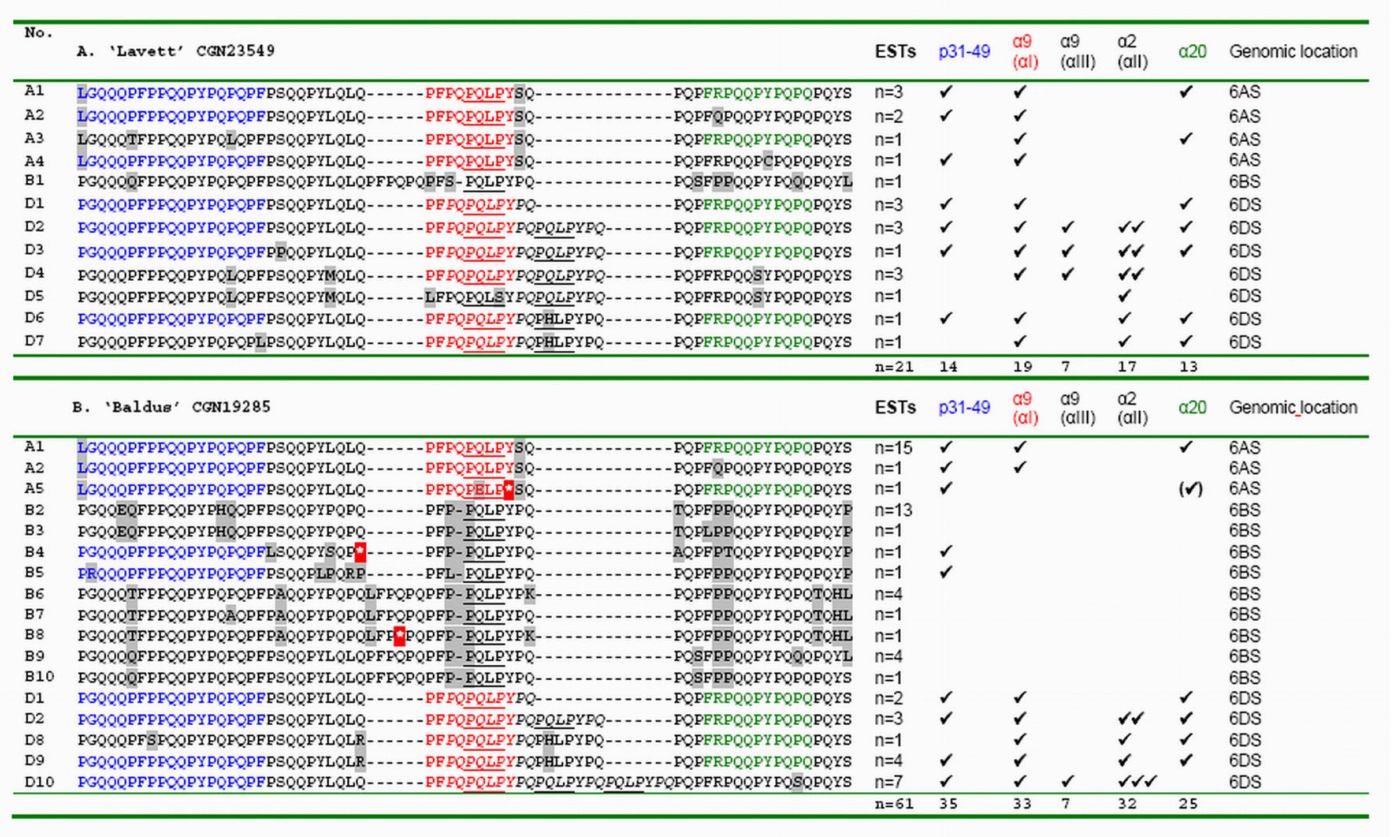
**Deduced α-gliadin isoforms and CD-epitope frequencies of cultivars**. Amino acid sequences of the N-terminal part of α-gliadins as deduced from α-gliadin transcripts present in developing grains at 14 days post anthesis (14DPA) of single genotypes derived from the respective cultivars 'Lavett' (**3A**), 'Baldus' (**3B**), This N-terminal locus contains several CD-epitopes: p31-49 (PGQQQPF*PP*QQ*P*YPQPQPF, in blue), triggering the innate immune response; and the HLA-DQ2+ restricted T-cell epitopes Glia-α9(αI) (PFPQPQLPY, in red); Glia-α9(αIII) (PYPQPQLPY); Glia-α2(αII) (PQPQLPYPQ) and Glia-α20 (FRPQQPYPQ, in green). The HLA-DQ2+ epitope frequency calculated as the number of conserved (ESTs) for 'Lavett', 'Baldus' is respectively 2.7 and 1.6. The consensus nucleotide sequences of the EST contigs were assigned to a predicted genomic location by clustering (Clustal W) with genomic sequences from diploid species with genome AA (*T. monococcum*), BB (SS) (*Aegilops speltoides*) and DD (*Aegilops tauschii*), as in Van Herpen et al. [14]. GenBank accession numbers of the ESTs: 'Lavett' GH160345-GH160365, 'Baldus' GH162284-GH160344.

**Figure 4:**
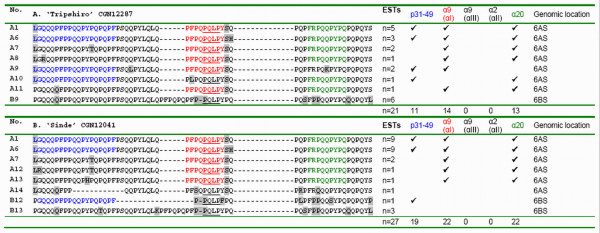
**Deduced α-gliadin isoforms and CD-epitope frequencies of landraces**. Amino acid sequences of the N-terminal part of α-gliadins as deduced from α-gliadin transcripts present in developing grains at 14 days post anthesis (14DPA) of single genotypes derived from the respective accessions of the landraces 'Tripshiro' (**4A**) and 'Sinde' (**4B**). This N-terminal locus contains several CD-epitopes: p31-49 (PGQQQPF*PP*QQ*P*YPQPQPF, in blue), triggering the innate immune response; and the HLA-DQ2+ restricted T-cell epitopes Glia-α9(αI) (PFPQPQLPY, in red); Glia-α9(αIII) (PYPQPQLPY); Glia-α2(αII) (PQPQLPYPQ) and Glia-α20 (FRPQQPYPQ, in green). The HLA-DQ2+ epitope frequency calculated as the number of conserved α9(αI), α9(αIII), α2(αII) and α20 T-cell epitopes per number of cDNA sequences (ESTs) for 'Tripshiro' and 'Sinde' is respectively 1.3 and 1.6. The consensus nucleotide sequences of the EST contigs were assigned to a predicted genomic location by clustering (Clustal W) with genomic sequences from diploid species with genome AA (*T. monococcum*), BB(SS) (*Aegilops speltoides*) and DD (*Aegilops tauschii*), as in Van Herpen et al. [14]. GenBank accession numbers of the ESTs: 'Tripshiro' GH160393-GH160413, 'Sinde' GH160366-GH160392.

By studying the deduced amino acid sequences the number of different isoforms and the frequency *Gli-2 *transcripts encoding conserved CD-epitopes were determined. In the 5' region (135 to 216 base pairs in size) of the *Gli-2 *transcripts analyzed here a total of 37 different α-gliadin isoforms were evident (Figure 3 and 4). The transcripts derived from the hexaploid genotypes derived from the cultivars 'Lavett' (Figure 3A) and 'Baldus' (Figure 3B) and the tetraploid genotypes derived from the landraces 'Tripshiro' (Figure 4A) and 'Sinde' (Figure 4B) encoded 12, 17, 8 and 8 different isoforms, respectively. These numbers are reflecting the minimal variation in α-gliadin isoforms expected to be present in the gluten fraction of the genotypes analyzed. Several isoforms were rare and only encoded by a single transcript, but some were highly expressed, which reflects their relative frequency in the gluten transcriptome. For instance, almost 25% of the α-gliadin transcripts (15 out of 61 ESTs) from 'Baldus' were encoding one isoform (no. A1, Figure 3B).

The *Gli-2 *fraction derived from the *Gli-B2 *locus had a reduced CD-immunogenic potential as compared to *Gli-A2 *and *Gli-D2 *α-gliadins (consistent with results of Molberg et al. and Van Herpen et al. [20,17]). Also, the *Gli-A2 *transcripts we obtained lack the sequence encoding the Glia-α2 epitope. In the hexaploid lines (expressing *Gli-D2 *genes) the average number of conserved HLA-DQ2+ CD-epitopes (Glia-α2 from *Gli-D2 *sequences; Glia-α9 and Glia-α20 from *Gli-A2 *and *Gli-D2 *sequences) per gliadin transcript was 2.7 for 'Lavett' and 1.6 for 'Baldus'. In the case of tetraploid lines, which express only *Gli-B2 *and *Gli-A2 *α-gliadins, the accessions with a low *Gli-A2 *expression frequency and thus the lowest number of conserved HLA-DQ2+ CD-epitopes in the α-gliadin transcriptome (in this example 'Tripshiro' with an average number of CD-epitopes per α-gliadin transcript of 1.3) are more interesting to test for low CD toxicity.

### A pyrosequencing assay to assess relative Gli-A2 expression frequencies

**Figure 5:**
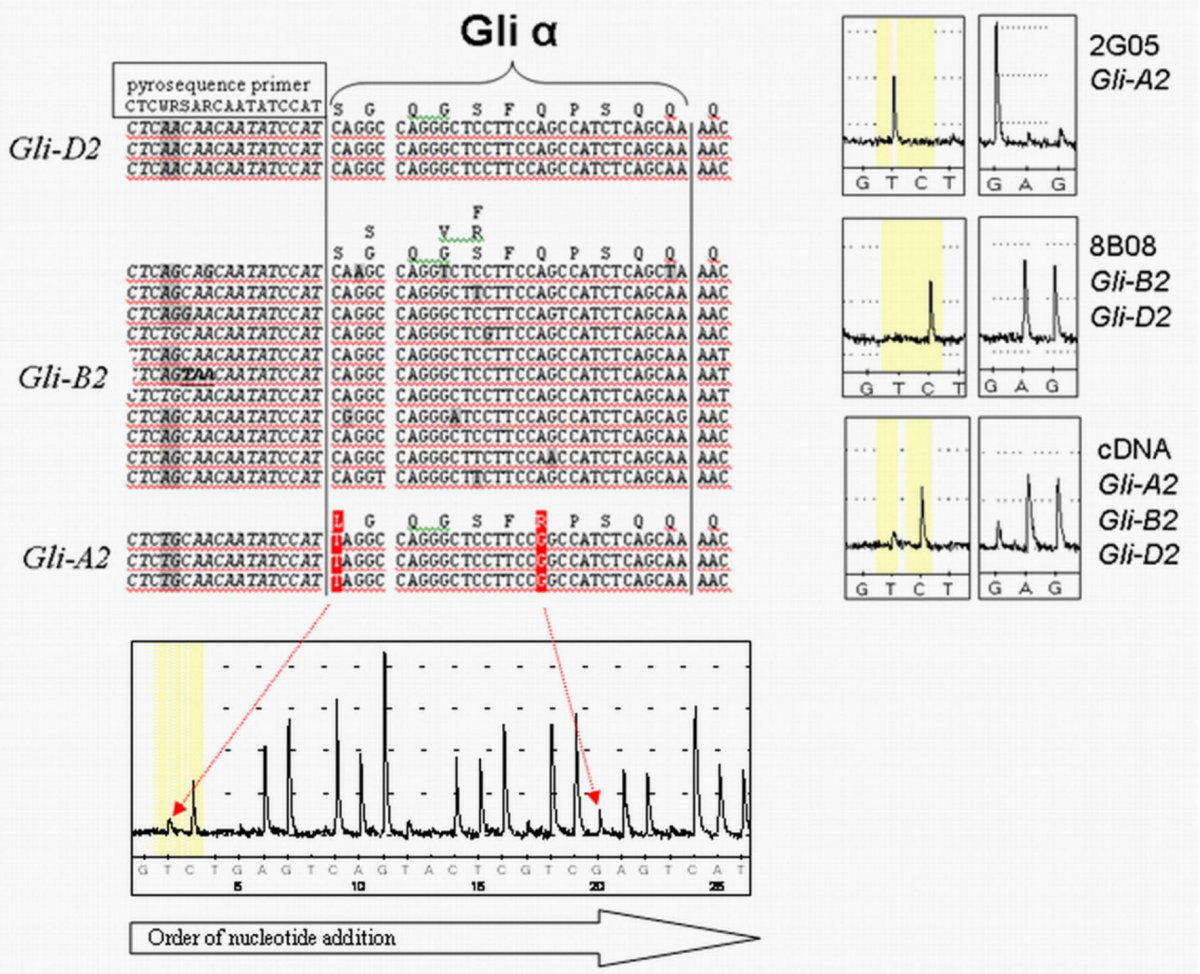
**Pyrosequencing assay specific for *Gli-A2 *sequences**. Pyrosequencing assay pointed towards SNPs linked to the A-genomic *Gli-2 *genes (*Gli-A2 *genes) of *Triticum *spp. (C/T and G/A, indicated in red). These SNP positions are located in the 3'-part of the gene in a sequence coding for a CD-epitope (Glia-α) conferring HLA-DQ8+ restricted T-cell stimulation.

The screening of wheat accessions for differential expression of *Gli-2 *genes from different homoeologous loci, varying in their CD-immunogenic potential, is a step towards the development of a wheat line with reduced CD-immunogenicity. As EST sequencing is too laborious for routine application in a breeding program, we developed a pyrosequening method to detect relative differences in the composition of the *Gli-2 *transcriptome among samples. This pyrosequencing method was developed based on DNA polymorphisms specific for *Gli-A2 *α-gliadins. Due to the large repetitive domains in each sequence and the large number of sequence variants only a few polymorphic sites in the gene were diagnostic for *Gli-A2*. We used the single nucleotide polymorphisms (SNPs) (T/C and GG/AG or AA) as indicated in Figure 5. The T (pyrogram position 2) and G (pyrogram position 20) nucleotides were found in all gliadin sequences derived from locus *Gli-A2*, whereas C (pyrogram position 3) and A (pyrogram position 21) nucleotides are present in all α-gliadin sequences derived from *Gli-B2 *or *Gli-D2 *loci. These two SNP positions are located in the 3' region of the gene, in the DNA sequence that codes for the Glia-α epitope. This epitope is toxic to patients that are HLA-DQ8+ and is conserved in *Gli-D2 *and *Gli-B2 *α-gliadin variants. In *Gli-D2 *isoforms it is physically linked to the more N-terminal HLA-DQ2+ restricted T-cell epitopes Glia-α9, Glia-α20, and Glia-α2 (Figure 1).

The specificity of the pyrosequencing assay for *Gli-A2 *sequences was verified in genomic DNA of the 'Chinese Spring' homozygous deletion line 6AS-1, which lacks two thirds of chromosome arm 6AS, including the *Gli-A2 *locus. This was clearly visible in the pyrograms of this deletion line in the form of the absence of the T- and G-nucleotide peaks, whereas the C- and A-nucleotide peaks, representing genes of both the *Gli-B2 *and *Gli-D2 *locus were always clearly detected (not shown). Both SNP positions (pyrogram position 2/3 and 20/21) returned similar *Gli-A2 *frequencies (F test, p = 0.067). When comparing the average *Gli-A2 *frequency of the two SNP positions in genomic DNA of two wild type wheat 'Chinese Spring' lines and deletion line 6AS-1 the pyrosequencing assay detected large differences in *Gli-A2 *frequency. Based on the replicate measurements in the genomic DNA of the 'Chinese Spring' lines, differences as small as 3.3% are detectable with this pyrosequencing assay (LSD = 3.3% ± 0.0 at F test, p < 0.001; general ANOVA in Genstat) (Table 2).

**Table 2:** *Gli-A2 *specific pyrosequencing in genomic DNA of 'Chinese Spring'

**Line**	**SNP1 (T2C3) mean**	**stdev**	**n**	**SNP2 (G20A21) mean**	**stdev**	**n**	**SNP1+SNP2 average**	**Differences** **P < 0.001**
**WT4086**	0.16	0.04	5	0.20	0.02	5	0.181	a
**WT4085**	0.16	0.05	5	0.21	0.03	5	0.185	a
**WT4084**	0.15	0.05	8	0.18	0.03	8	0.165	a
**6AS-1**	0.00	0.02	5	0.00	0.01	5	0.000	b

Frequency of α-gliadin gene copies derived from the *Gli-A2 *locus in genomic DNA of three 'Chinese Spring' accessions and in the homozygous deletion line 6AS-1 of 'Chinese Spring' missing the short arm of chromosome 6A, as determined by pyrosequencing on two SNP positions (T2/C3 & GG20/A21), n = number of technical repetitions performed per 'Chinese Spring' line.; a, b: a different letter indicates a statistically significant difference between the two samples; LSD for the *Gli-A2 *frequency among 'Chinese Spring' lines = 3.3% ± 0.004 at P < 0.001 (general ANOVA; Genstat).

### The Gli-A2 expression frequency during grain development

To study α-gliadin expression from the *Gli-A2 *locus during grain development, grains from two bread wheat cultivars ('Baldus' and 'Lavett') and one tetraploid durum wheat cultivar ('Probstdorfer Pandur') were sampled at 7, 14 and 21 days after anthesis (DPA). Van Herpen et al. [25] showed that the α-gliadin promotor is active during endosperm development, but did not quantify α-gliadin expression levels during development. In cDNA samples of the three developmental stages analyzed we observed that the *Gli-2 *transcripts were clearly detectable as RT-PCR products in approximately similar amounts (results not shown). As measured by pyrosequencing, the *Gli-A2 *transcript frequency in the α-gliadin transcriptome of the durum wheat cultivar 'Probstdorfer Pandur' was 41% on both harvest dates. In the hexaploid cultivars 'Baldus' and 'Lavett', which in addition also transcribe *Gli-D2 *genes, we measured a lower *Gli-A2 *transcript frequency, ranging from 10% to 21%. The transcript frequency of *Gli-A2 *gliadins showed no obvious differences during grain development from 7 DPA to 21 DPA (Table 3). As these are samples from a few plants from only one cultivation year, we do not know whether these frequencies are consistently present. Considering the cDNA samples of all wheat accessions and all harvest dates together, no significant effect of the harvest date on the *Gli-A2 *frequency was found (ANOVA, F test, p = 0.732), whereas the ploidy level does have a significant effect (ANOVA, F test, p < 0.001). Based on the replicate measurements in two 'Baldus' genotypes (Table 3), differences as small as 5.9% are detectable in cDNA samples with this pyrosequencing assay (LSD = 5.90% ± 0.02 at F test, p < 0.001; general ANOVA in Genstat).

**Table 3:** *Gli-A2 *transcript frequency during grain development

**Accession**	**ploidy**	**7DPA**	**n**	**14DPA**	**n**	**21DPA**	**n**
Baldus CGN19285	Hexaploid	0.21 ± 0.01	2	0.19 ± 0.04	4	0.17 ± 0.03	4
Lavett CGN23549	Hexaploid	0.10 ± 0.07	1	0.13 ± 0.02	1	0.13 ± 0.08	2
Probstdorfer Pandur CGN08262	Tetraploid	nd	0	0.41 ± 0.05	1	0.41 ± 0.04	1

Frequency of *Gli-A2 *expression in the hexaploid *T. aestivum *cultivars 'Baldus' and 'Lavett' (AABBDD genome) and the tetraploid *T. turgidum *cultivar 'Probstdorfer Pandur' (AABB genome) as determined with pyrosequencing in cDNA fractions obtained from different stages of grain development (7, 14, and 21 days post anthesis (DPA)). The frequencies were calculated as the average of 1–4 different cDNA samples (n) at two different SNP positions, T2/(T2+C3) and G20/(G20+A21); ± = standard deviation for measurements, including both SNP positions.

### The Gli-A2 expression frequency in different wheat accessions

To pre-select wheat accessions that may differ in the composition of the α-gliadin fraction and in CD-immunogenic potential, individual plants derived from sixteen different wheat accessions were assessed by pyrosequencing for the *Gli-A2 *transcript expression frequency in developing wheat kernels. To include more genetic variation the accessions used originated from different geographical locations. Furthermore, three different population types were included: advanced cultivars, selections used for breeding, and landraces. Advanced cultivars ('Baldus', 'Tripshiro' and 'Probstdorfer Pandur') and selections used for breeding (CGN10569 and 'Dakar 52') are genetically more homogeneous compared to landraces, which are often mixtures of distinct genotypes that may even differ in ploidy level (personal communication Dr. Noortje Bas and Van den Broeck et al., in prep). Information on population type and origin is listed in Table 1. The advanced tetraploid cultivar was included to assess the variation in *Gli-A2 *expression in a situation without *Gli-D2 *homoeologs. In this situation, highly CD-immunogenic sequences originating from *Gli-D2 *are absent [20] and because *Gli-B2 *copies harbour less CD-epitopes [17] the CD-immunogenicity mainly comes from expression of *Gli-A2 *isoforms.

**Figure 6:**
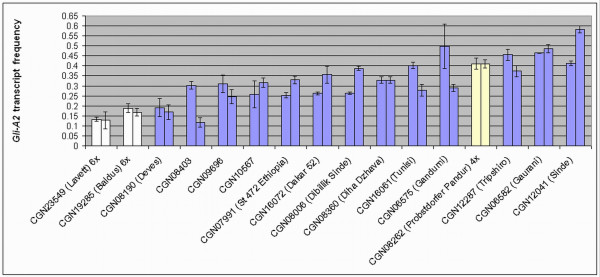
***Gli-A2 *transcript frequency in different wheat genotypes**. The *Gli-A2 *frequency in the total α-gliadin transcriptome of developing seeds (cDNA). Among the accessions are advanced cultivars of bread wheat (*T. aestivum *L.) (in white), a durum wheat (*T. turgidum *var. durum) cultivar (in yellow), an accession used for breeding research (CGN10567) and more genetically diverse landraces (in purple). For each accession, the *Gli-A2 *frequency in the cDNA fractions of two individual plants was measured with pyrosequencing on two SNP positions C2/C2+T3 and G20/G20+A21. Bars indicate the standard deviation for the technical replications. The advanced cultivars are either registered as tetraploid (4×) or hexaploids (6×). Landraces (blue bars) may be mixtures of both ploidy levels and therefore the ploidy level of the individuals derived from a landrace accession is uncertain. More information about the accessions is given in Table 1.

The *Gli-A2 *transcript frequencies that were obtained by pyrosequencing are shown in Figure 6 for two individual plants per accession. The relative *Gli-A2 *expression frequencies for individual genotypes ranged from 12% for a plant from CGN08403 to as much as 58% for a genotype of the landrace 'Sinde' (CGN12041). In the advanced hexaploid cultivars 'Baldus' and 'Lavett' the highest *Gli-A2 *expression frequency observed was 13% for 'Lavett' and 19% for 'Baldus' (Figure 6, white bars). In the tetraploid plants, in which there is no *Gli-D2*, the *Gli-A2 *expression frequency reached relative higher levels. For instance, the relative *Gli-A2 *expression frequency of the durum wheat cultivar 'Probstdorfer Pandur' was 41% (Figure 6, yellow bar).

To investigate the possibility for *Gli-A2 *expression frequency analysis in genetically more heterogeneous material several landraces were included (Figure 6, blue bars). Here the *Gli-A2 *expression frequency of individual genotypes ranged from 12% to 58% and in most cases significant variation was observed within accessions. Most individual genotypes displayed *Gli-A2 *expression frequencies which were intermediate between the hexaploid (white bars; Figure 6) and tetraploid (yellow bars, Figure 6) situation. Depending on their actual ploidy level these genotypes are interesting candidates for further analysis towards the selection of low CD-immunogenic genotypes. In case of a hexaploid genome, a significantly higher *Gli-A2 *expression is desired (as it implies a lower expression of the highly CD-immunogenic *Gli-D2 *gliadins [20,17]). On the contrary, tetraploid genotypes require a low *Gli-A2 *expression to reduce the already lower CD-immunogenicity of their gluten (as *Gli-A2 *gliadins contain more CD epitopes than the very low CD-immunogenic *Gli-B2 *sequences [17]).

## Discussion

The most immunogenic fraction of gluten with respect to celiac disease (CD) is the fraction of α-gliadin proteins. Differences in T-cell stimulation were observed among gluten (gliadin) extracts of different wheat varieties [19]. However, the analysis of CD-immunogenicity of individual wheat varieties by *in vitro *T-cell clones remains time consuming and is sensitive to the quality and specificity of individual T-cell clones. In addition, T-cell tests are qualitative rather than quantitative. To test large numbers of wheat lines for differences in CD-immunogenicity, T-cell independent methods are highly desired. Naturally occurring single amino acid changes in the sequences of CD-epitopes may cause a significant reduction in the level of *in vitro *T-cell stimulation [26]; [Mittea et al. in preparation, Salentijn et al. in preparation], which is on its turn a reflection of clinical relevance. Furthermore, it has been shown that T-cells recognizing imperfect CD-epitopes are less frequently present in peripheral blood of CD patients [27,28]. As the variant CD-epitopesexpressed from the *Gli-B2 *locus lack perfect HLA-DQ2+ T-cell epitopes [17], a relatively high level of α-gliadin expression from this locus is most desired from the perspective of generating wheat varieties with a reduced CD-immunogenic capacity. A tetraploid variety with only *Gli-B2 *expression would contain hardly any α-gliadin CD-epitopes. In hexaploid varieties, in addition to a high expression from the *Gli-B2 *locus, a higher *Gli-A2 *(low *Gli-D2*) frequency is relatively better, as this reduces the level of the most immunogenic *Gli-D2 *isoforms.

Each of the homoeologous genomic loci, *Gli-A2*, *Gli-B2 *and *Gli-D2*, controls the synthesis of a group (block) of gliadin proteins that are jointly inherited as a Mendelian unit (alleles). These allelic blocks differ in the number, the intensity, and the electrophoretic mobility of gliadin bands [29]. Only a few gliadin-coding genes were reported as remote from the gene clusters and able to recombine with them (referred to as 'selfish', 'removed' genes or 'additional' gliadin loci) [e.g. [30]]. The allelic blocks were used to describe genetic diversity in *T. aestivum *and *T. turgidum *wheat varieties originating from specific geographic regions and a wide variation was found in the one- and two-dimensional electrophoretic patterns of gliadins from both bread and durum wheat genotypes, including inter-varietal polymorphism, the occurrence of biotypes within cultivars, alleles specific for a region, and intermixing and duplication of gene bank accessions [29,31-34]. A high level of genetic variation was confirmed here in a 5' sequence of α-gliadins coding for CD-epitopes. Α-gliadin gene transcripts from two bread wheat cultivars and two landraces of wheat together encoded 37 different α-gliadin isoforms (14 *Gli-A2 *isoforms, 13 *Gli-B2 *isoforms and 10 *Gli-D2 *isoforms). These isoforms were found in a small region (135 to 216 base pairs in size) in the 5' part of the α-gliadin gene and therefore reflect only part of the variation within these four genotypes. In a Spanish collection of 22 cultivated diploid einkorn wheats, which contain the A^m^-genome (diploid, *T. monococcum *L. spp. *monococcum*), 14 different *Gli-A2 *allelic blocks were found, with up to four different alleles per accession. The allele diagrams were composed of a number of 5 up to 8 principle protein bands [35]. Here we observed a similar number of *Gli-A2 *isoforms per genotype ranging from 3 to 10 (Figure 3 and 4).

Several studies have shown the unequal transcription from the three homoeologous loci in hexaploid wheat [36,37]. Nomura et al. [38] found differential expression among the three homoeologous Bx-genes involved in benzoxazinone biosynthesis and demonstrated a bias in transcript levels towards the Bx homoeologs of the B genome. Some of the alleles were efficiently transcribed but showed only a weak enzymatic activity with the genes from the B-genome contributing the most to Bx biosynthesis. Recently, Shitsukawa et al. [39] found a differential contribution of the three homoeologs of a wheat class E MADS box gene (WLHS1), where the A genome WLHS1 homoeolog appeared to be inactivated by an insertion and the B homoeolog was predominantly silenced by cytosine methylation. Kawaura et al. [21] observed that *Gli-B2 *transcripts were underrepresented compared to their homoeologs from the A and D genomes in a set of ESTs from the NCBI database, which was a mixture of sequences obtained from 'Chinese Spring' and some other genotypes. Here, we confirm differences in relative expression of the homoeologous α-gliadin genes among genotypes, both as relative numbers of α-gliadin transcript sequences from specific varieties as well as by using a quantitative pyrosequencing assay for detection of relative differences in *Gli-A2 *transcript frequencies larger than 5.9%.

Detailed sequence analysis of cloned α-gliadin transcripts showed that in some hexaploid varieties the *Gli-B2 *genes were hardly expressed (e.g., less than 5% in 'Lavett', Figure 2) but in others they made up more than 40% (in 'Baldus', Figure 2).

As a consequence, the predicted CD-immunogenic capacity is partly depending on the ratio of differential expression of the homoelogous *Gli-2 *genes. These differences in homoeoallelic expression may be due to epigenetic factors, or to the relative efficiency of transcription factors to stimulate transcription across the homoeologous promoters in the polyploid. In the latter case, the factors could act partly *in cis *(promoters that are switched on more easily or are very specific) or *in trans *(differences in promiscuity of transcription factors from the three genomes). Studies of relative expression levels in progeny of crosses may answer some of these questions. For this, a method for the rapid and high throughput determination of differences in allele frequencies of single nucleotide polymorphisms in pools of DNA would be convenient, and according to Neve et al. [40] and Wasson et al. [41] pyrosequencing is a suitable method for this purpose. SNP allele frequencies with 4% difference between populations could reliably be detected [27] and for large genomic DNA pools, allele frequencies that differed by more then 5.2% would be significant [41]. Schaart et al. [42] used pyrosequencing to study differences in the expression level of six different, but highly (96.3% to 99.8%) homologous alleles of a pathogenesis-related gene (PGIP) in octoploid strawberry (*Fragaria *× ananassa). For genomic DNA samples, PGIP gene differences in allele frequencies as small as 4.0 ± 2.8% were detected while for cDNA samples a higher variation was observed between the repeats in the experiments, ranging from 7.8 ± 1.3% for leaf and fruit samples to 10 ± 2.1% in an experiment that included inoculation with the fungus *Botrytis cinerea*. In these experiments different PGIP alleles that were specifically expressed in leaf and fruit tissue were identified. These results demonstrated that pyrosequencing of cDNA samples is a useful method to determine allele expression frequencies.

For the complex *Gli-2 *locus, we have developed here a pyrosequencing assay based on two SNPs that are specific for *Gli-A2 *genes. Somers et al. [43] proposed to call such nucleotide polymorphisms 'homoeologous sequence variants' (HSV) as they do not distinguish alleles that are inherited in a Mendelian fashion, but rather are sequence variants that may occur in the same haploid genome. In genomic DNA of hexaploid wheat cultivar 'Chinese Spring' our assay detected significant frequency differences for the *Gli-A2 *genes as small as 3.300% ± 0.004 at P < 0.001 (general ANOVA in Genstat, Table 2), which is in the expected range for genomic DNA samples [40-42]. Based on the replications in 'Baldus' (Table 3), differences in *Gli-A2 *transcript frequency as small as 5.9% are detectable among cDNA samples with this pyrosequencing assay (LSD = 5.90% ± 0.02 at F test, p < 0.001; general ANOVA in Genstat). The larger variation in *Gli-A2 *frequency in cDNA samples might be due to fluctuations in expression of *Gli-2 *genes induced by environmental or physiological factors. As the plants were grown in the same season on plots in the same field these differences were most likely not caused by the environment but may reflect the high level of genetic diversity among the plants within wheat accessions from the genebank (see below).

The relative differences in frequency of *Gli-A2 *expression observed in the cloned expressed sequence tag (EST) sequences of various genotypes (Figure 2) and those found with pyrosequencing of *Gli-A2 *SNPs (Figure 6) correlated well in a comparison including tetraploid and hexaploid genotypes (R^2 ^= 0.96). Pyrosequencing was not sensitive enough for the detection of relative differences in *Gli-A2 *transcript frequency below 5.9%. Also, the absolute levels of *Gli-A2 *transcripts were always higher in the EST dataset. The amplicon used for cloning and sequencing of all ESTs is located in the 5'-region of the α-gliadins. As the *Gli-2 *transcriptome is complex and may possibly include over 100 different gene copies with a low overall homology (<60%), and we were restricted to domains that are conserved within each of the homoeologous loci for the development of primers for pyrosequencing, these primers were designed in the 3'-region of the genes, and they had to include some degeneracy in the sequences. It is therefore possible that a part of the *Gli-A2 *copies might not be targeted by the pyrosequence primers, which would account for some underestimation of the A-genome sequences. An alternative explanation is that part of the *Gli-A2 *genes would lack the specific SNPs located in the 3'-part of the gene while they are homologous to A-genomic α-gliadins in the 5'-region. However, this is not likely as such variants have not been found in our EST study, or in the genomic clones analyzed by Van Herpen et al. [17]. We are currently developing a new pyrosequence assay that returned approximately twofold higher *Gli-A2 *allele frequencies (results not shown).

Many genebank accessions and cultivars are not composed of genetically uniform germplasms [34,44,45]. Accessions of landraces are most often a mixture of hexaploid *T. aestivum *and tetraploid *T. turgidum *group *durum *genotypes, as was evident for 'Sinde' CGN12041 in this study. The accession was initially determined as hexaploid *T. aestivum *(AABBDD) but the individual genotype analyzed here expressed no *Gli-D2 *sequences suggesting this plant was a tetraploid (AABB). Indeed, recently it has been observed that hexaploid and tetraploid individuals of some Northern African landraces display a striking resemblance in their appearance facilitating the mixing of both genotypes (personal communication Dr. Anton Zeven and Dr. Noor Bas, CGN). The land variety CGN08190 (Deves) is another example of the mixed nature of some genebank accessions. This accession is registered as tetraploid durum wheat but after analyzing expressed sequence tag sequences it turned out that some plants of this accession contained *Gli-D2 *transcripts derived from hexaploid wheat (results not shown). The relative *Gli-A2 *expression frequencies of 17% and 19% as returned by pyrosequencing (Figure 6) are comparable to the frequencies found in bread wheat cultivars (which range between 13% and 19%).

As determined by pyrosequencing several *T. turgidum *accession have genotypes with a lower *Gli-A2 *transcript frequency compared to the advanced durum wheat cultivar 'Probstdorfer Pandur' (<41% *Gli-A2 *frequency) and are interesting to include in studies towards the development of low CD-immunogenic tetraploid wheat varieties; for instance, 'Dakar 52' (26% and 36% *Gli-A2 *transcripts) and the landraces CGN07991 (25% and 33% *Gli-A2 *transcripts) and 'Dibillik Sinde' (26% and 39% *Gli-A2 *transcripts). The breeding line CGN10567 (26% and 32% *Gli-A2 *transcripts) is an interesting hexaploid candidate to include in further analysis. For the selection of individual genotypes with reduced CD-immunogenic potential individual genotypes of these accessions need to be characterized for ploidy level and for the frequency of sequences coding for CD-epitopes in the *Gli-2 *transcriptome. Next, the gluten fractions of selected plants have to be subjected to immunoblot analysis using antibodies raised against CD-epitopes and *in vitro *T-cell tests. The pyrosequence assay developed will be a useful tool in the preliminary screening of *Gli-A2 *frequencies wheat genotypes derived from a wide range of wheat accessions. Furthermore, since α-gliadins are not the only epitope-containing fraction, subsequent studies on selected lines should incorporate assays to screen for variation in other immunogenic gluten peptides that are present in γ-gliadins and low molecular weight glutenins. Future research may use new generation DNA sequencing technologies [46,47] to enable high throughput analysis of pre-selected wheat lines for variation in CD-epitopes.

## Conclusion

Here, we have shown that large differences exist in relative expression levels from homoeologous *Gli-2 *loci among wheat genotypes, both as relative numbers of ESTs from specific varieties (Figure 3 and 4) and when using a quantitative pyrosequencing assay specific for *Gli-A2 *α-gliadin genes (Table 3, Figure 6). The *Gli-A2 *specific pyrosequence assay was used to screen plants derived from a series of wheat varieties for differential allelic expression levels (>5.9%). The relative *Gli-A2 *expression level in a tetraploid durum wheat cultivar ('Probstdorfer Pandur') was 41%. In hexaploid bread wheat cultivars the relative *Gli-A2 *frequency varied between 13% and 19% and in landraces between 12% and 58%. The detailed analysis of ESTs showed that in plants derived from two hexaploid bread wheat cultivars the expression level of α-gliadin genes from locus *Gli-B2*, encoding low CD-immunogenic α-gliadin isoforms, ranged from less than 5% up to more than 40% of the α-gliadin transcripts. Screening for differential *Gli-A2 *expression can be employed for the pre-selection of wheat varieties in the search for varieties with very low CD-immunogenic potential.

## Methods

[GenBank: GH162284 – GH160413; dbESTid: 63157496-63157625]

### Plant material

The homozygous 'Chinese Spring' deletion line 6AS-1 (Ta4540 L1), which lacks a part of the short arm of chromosome 6A including the *Gli-A2 *locus, was obtained from the Wheat Genetic and Genomic Resources Center . This deletion line was used to test the A-genome specificity of the pyrosequence assay. The hexaploid (genome composition AABBDD) 'Chinese Spring' lines CGN04084, CGN04085 and CGN04086 were used as controls. The hexaploid cultivars 'Baldus' (CGN19285) and 'Lavett' (CGN23549) and the tetraploid (AABB) cultivar 'Probstdorfer Pandur' (CGN08262) were assayed for the frequency of the *Gli-A2 *sequences in the α-gliadin transcriptome at three developmental stages; 7 days post anthesis (DPA), 14 DPA and 21 DPA. These and other wheat accessions (Table 1) were obtained from the Centre for Genetic Resources (CGN), the Netherlands . Those listed in Table 1 were assayed for *Gli-A2 *frequency in cDNA of developing wheat grains by pyrosequencing. The plants were grown in adjacent fields in the spring and summer of 2005 at location the 'Kievit', Plassteeg, Wageningen, The Netherlands in a sandy soil. The field was fertilized with Tripelsuperfosfate (45% P_2_O_5_) 108.97 kg/ha, Kali60 (60% K_2_O) 108.97 kg/ha, Kalkammonsalpeter (27% N; NH_4_NO_3 _+ 6% CaCO_3_) 275 kg/ha. Developing kernels of two individual plants per accession were harvested at 14DPA and 21DPA.

### DNA isolation, RNA isolation and cDNA synthesis

Both, DNA and RNA were isolated according to the method described by Doyle and Doyle [48] but with 1% (w/v) poly-(vinylpyrrolidone)-10 in the extraction buffer. Genomic DNA of the deletion line 6AS-1 and 'Chinese Spring' lines was isolated from young leaves of 4 to 5 seedlings per genotype. Total RNA fractions of wheat accessions were isolated from grain tissue of 3–4 grains of one ear of single plants, grown in adjacent fields in the spring and summer of 2005. The ears were harvested at 7, 14 and 21 DPA. For the production of first strand cDNA 1 μg of total RNA was treated with DNAse I (Invitrogen, amplification grade; 18068-015) followed by RT PCR (Invitrogen SuperScript™ III First-Strand Synthesis System for RT-PCR; 18080-051) using random hexamer primers in a final reaction volume of 20 μl. Samples without SuperScript™ III reverse transcriptase (minus RT PCR) were included as controls for the DNAse I treatment.

### cDNA analysis

A fraction of 2 μl of the cDNAs was used as a template in a PCR reaction with α-gliadin-specific primers (α1F: 5'atg aaR acM ttt cYc atc-3'; α5R; 5'gtt agt acc gaa gat gcc-3'). Subsequently, the PCR products were cloned in pGEM^®^-T Easy (Promega). For 'Tripshiro', 'Sinde', 'Lavett' and 'Baldus' the 5'-part of respectively 21, 27, 21 and 61 α-gliadin cDNA clones was sequenced. [GenBank accession numbers: GH160393-GH160413 ('Tripshiro'), GH160366-GH160392 ('Sinde'), GH160345-GH160365 ('Lavett'), GH162284-GH160344('Baldus')]

This part of the gene contains the sequences coding for important HLA-DQ2+ CD-epitopes, Glia-α2(αII), Glia-α9(αI), Glia-α9(αIII), Glia-α20 and an epitope involved in the innate CD-immune response, p31-49 (Figure 1). The sequences were assigned to one of the homoeologous *Gli-2 *loci (*Gli-A2*, *Gli-B2 *or *Gli-D2*) by clustering (Clustal W) with genomic sequences from diploid species *Triticum monococcum *(A-genome; DQ002569 to DQ002583), *Aegilops speltoides *(B-genome, DQ002584-DQ002588) and *Aegilops tauschii *(D-genome; DQ002589-DQ002599), as in Van Herpen et al. [17].

### Pyrosequencing

First, a pyrosequence assay was developed using a primer set (α-3prime-F1/α-3prime-R1-biotin) to amplify the 3' target fragment for pyrosequencing, including all *Gli-2 *genes, from 20 ng of genomic DNA (deletion line 6AS-1 and Chinese Spring) or 2 μl cDNA sample. This amplification was performed in a 50 μl reaction volume, containing 0.4 μM of both reverse and forward primers (α-3prime-F1: 5'-cag***Y***ctc***WRB***a***R***caatatcc-3'; α-3prime-R1: 5'-Biotin-tggagggat***R***ta***B***acattgc-3'; both were HPLC-purified), dNTP mix (0.2 mM each), 1.5 mM of MgCl_2_, 1× Goldstar buffer (Eurogentec), and 1 U Goldstar DNA polymerase (Eurogentec) (5 U/μl). By performing 50 cycles of PCR all primers are used up to prevent interference with pyrosequencing. PCR cycling used for cDNA: denaturation at 94°C for 5 minutes followed by 50 cycles of {94°C for 30 seconds, 51°C for 1 minute and 72°C for 2 minutes} and 72°C for 10 minutes. PCR cycling used for genomic DNA: denaturation at 94°C for 5 minutes followed by 50 cycles of {94°C for 30 seconds, 48°C for 1 minute and 72°C for 2 minutes} and 72°C for 10 minutes. The PCR product (30 μl) was linked to streptavidine sepharose HP beads (Amersham Biosciences) by incubation for 10 minutes at room temperature while shaking (4 μl beads in 26 μl 10 mM TRIS-HCl; 2 M NaCl; 1 mM EDTA; 0.1% Tween 20).

Subsequently, the biotinylated PCR product was isolated using a Vacuum prep tool, washed in 70% ethanol for 5 sec, denatured in 0.2 M NaOH for 5 sec and neutralized for 5 sec in 10 mM Tris-acetate, pH = 7.5. Next, the biotinylated strand was transferred to 45 μl primer solution (0.3 μM primer in 20 mM Tris-acetate, 2 mM MgAc_2_). To target as many different *Gli-A2 *transcripts as possible in one cDNA or genomic DNA sample a degenerated primer was used for pyrosequencing. This primer consisted of the primers α3'-SQ1a to α3'-SQ1e mixed in equally amounts (0.06 μM each):

α3'-SQ1a 5'-CTC**T**GCAACAATATCCAT-3';

α3'-SQ1b 5'-CTCAGCAACAATATCCAT-3';

α3'-SQ1c 5'-CTCAG**G**AACAATATCCAT-3';

α3'-SQ1d 5'-CTCAGCA**G**CAATATCCAT-3';

α3'-SQ1e 5'-CTCA**A**CAACAATATCCAT-3'.

The pyrosequencing reaction was performed in a PSQ96MA (Biotage (formerly Pyrosequencing) AB, Uppsala, Sweden) with the following specific nucleotide dispensation order; GTCTGAGTCAGTACTCGTCGAGTCATCTCAGCTAG.

### Calculation of the allele frequencies

The frequency of the *Gli-A2 *transcripts, expressed from the A-genome, was calculated as follows:

1. Normalization of peak heights: Each sample was normalized for the background signal by subtracting the zero peak values from the peak. Subsequently, the resulting peak heights were calculated relative to a defined reference peak (peak A on position 10) corresponding to a non-variable position in the sequence.

2. The *Gli-A2 *frequency was calculated from the normalized peak heights on two SNP positions (SNP1 and SNP2). For SNP1 the frequency was calculated as the height of peak position 2 (a T residue on pyrogram position 2 is specific for *Gli-A2 *genes) relative to the total peak height for this position, which is the signal at position 2 plus that at position 3 (C on position 3 is specific for *Gli-B2 *and *Gli-D2 *genes). For SNP2 the *Gli-A2 *frequency was calculated as the height of peak position 20 (a G on position 20 is present in all *Gli-A2 *genes) relative to the total peak height for the SNP position 20 + position 21 (A on position 21 is specific for *Gli-B2 *and *Gli-D2 *genes). Per sample at least three pyrograms were generated and the average frequency returned by both SNPs was calculated. For the analysis of the *Gli-A2 *frequency in the *Gli-2 *transcriptome of wheat accessions, the analysis was performed in triplicate on replicate samples of cDNA harvested on 21DPA and 14DPA, and in some cases also on 7DPA.

3. In case that the *Gli-A2 *frequency was determined in comparison to a reference sample, the standard deviation (s) of the resulting difference was calculated from standard deviations (s) of the samples using the formula s = √(s1^2 ^+ s2^2^)

4. To test for differences among accessions, for each accession at least two replicates (14DPA and 21DPA) were assessed in an ANOVA (Genstat_general ANOVA) and in case of significant differences (P < 0.001), all comparisons were also tested in Fishers' unprotected least significant difference test.

## Authors' contributions

EMJS and MJMS designed the study. NB gave advice on the selection of plant material and cultivated it. EMJS harvested the material and carried out the sequence analysis. EMJS and SVG developed the pyrosequencing assay. SVG and TB carried out the cDNA analysis and contributed to the optimization of the pyrosequence assay. EMJS, MJMS and LJWJG drafted the manuscript with assistance of IMM and HCB. All authors read and approved the final manuscript.

## References

[B1] Huang S, Sirikhachornkit A, Su X, Faris J, Gill B, Haselkorn R, Gornicki P (2002). Genes encoding plastid acetyl-CoA carboxylase and 3-phosphoglycerate kinase of the Triticum/Aegilops complex and the evolutionary history of polyploid wheat. Proc Natl Acad Sci USA.

[B2] Feldman M, Lupton FGH, Miller TE, Smartt J, Simmonds NW (1995). Wheats. "Evolution of Crop Plants".

[B3] Shewry PR, Tatham AS, Shewry PR, Casey R (1999). The characteristics, structures and evolutionary relationships of prolamins. "Seed Proteins".

[B4] Shewry PR, Halford NG, Lafiandra D (2003). Genetics of Wheat Gluten Proteins. Adv Genet.

[B5] Rewers M (2005). Epidemiology of celiac disease: what are the prevalence, incidence, and progression of celiac disease?. Gasteroenterology.

[B6] Koning F, Gilissen L, Wijmenga C (2005). Gluten: a two-edged sword. Immunopathogenesis of celiac disease. Springer Semin Immunopathol.

[B7] Koning F, Schuppan D, Cerf-Bensussan N, Sollid LM (2005). Pathomechanisms in celiac disease. Best Pract Res Clin Gastroenterol.

[B8] Diosdado B, Wijmenga C (2005). Molecular mechanisms of the adaptive, innate and regulatory immune responsese in the intestinal mucosa of celiac disease patients. Expert Rev Mol Diagn.

[B9] Ciccocioppo R, Di Sabatino A, Corazza GR (2005). The immune recognition of gluten in coeliac disease. Clin Exp Immunol.

[B10] Hüe S, Mention JJ, Monteiro RC, Zhang S, Cellier C, Schmitz J, Verkarre V, Fodil N, Bahram S, Cerf-Bensussan N, Caillat-Zucman S (2004). A direct Role for NKG2D/MICA Interaction in villous atrophy during celiac disease. Immunity.

[B11] Stepniak D, Koning F (2006). Celiac Disease-Sandwiched between Innate and Adaptive Immunity. Hum Immunol.

[B12] Payne PI, Lawrence GJ (1983). Catalogue of alleles for the complex gene loci, *Glu-A1*, *Glu-B1*, and *Glu-D1 *which code for high-molecular-weight subunits of glutenin in hexaploid wheat. Cereal Res Commun.

[B13] Gu YQ, Crossman C, Kong X, Luo M, You FM, Coleman-Derr D, Dubcovsky J, Anderson OD (2004). Genomic organization of the complex α-gliadin gene loci in wheat. Theor Appl Genet.

[B14] Harberd NP, Bartels D, Thompson RD (1985). Analysis of the gliadin multigene locus in bread wheat using nullisomic-tetrasomiclines. Mol Gen Genet.

[B15] Okita TW, Cheesbrough V, Reeves CD (1985). Evolution and heterogeneity of the alpha/beta type and gamma-type gliadin DNA sequences. J Biol Chem.

[B16] Anderson OD, Litts JC, Greene FC (1997). I. Characterization of ten new wheat α-gliadin genomic clones, evidence for limited sequence conservation of flanking DNA, and southern analysis of the gene family. Theor Appl Genet.

[B17] Van Herpen TWJM, Goryunova SV, Schoot J van der, Mitreva M, Salentijn EMJ, Vorst O, Schenk MF, van Veelen PA, Koning F, van Soest LJM, Vosman B, Bosch D, Hamer RJ, Gilissen LJWJ, Smulders MJM (2006). Alpha-gliadin genes from the A, B, and D genomes of wheat contain different sets of celiac disease epitopes. BMC Genomics.

[B18] Anderson OD, Greene FC (1997). The α-gliadin gene family. II DNA and protein sequence variation, subfamily structure and origins of pseudogenes. Theor Appl Genet.

[B19] Spaenij-Dekking L, Kooy-Winkelaar Y, van Veelen R, Drijfhout JW, Jonker H, van Soest L, Smulders MJM, Bosch D, Gilissen LJWJ, Koning F (2005). Natural variation in toxicity of wheat: potential for selection of non-toxic varieties for celiac disease patients. Gastroenterology.

[B20] Molberg Ø, Uhlen AK, Jensen T, Solheim Flaete N, Fleckenstein B, Arentz-Hansen H, Raki M, Lundin KEA, Sollid LM (2005). Mapping of Gluten T-Cell Epitopes in the Bread Wheat Ancestors: Implications for Celiac Disease. Gastroenterology.

[B21] Kawaura K, Mochida K, Ogihara Y (2005). Expression Profile of Two Storage-Protein Gene Families in Hexaploid Wheat Revealed by Large-Scale Analysis of Expressed Sequence Tags. Plant Physiol.

[B22] Tohver M (2007). High molecular weight (HMW) glutenin subunit composition of some Nordic and Middle European wheats. Genet Res Crop Evol.

[B23] Vallega V (1988). High molecular weight glutenin subunit composition of 115 cultivars of Triticum turgidum var. durum from various origins. Genet Agr.

[B24] Ma Z-C, Wei Y-M, Yan Z-H, Zheng Y-L (2007). Characterization of α-gliadin genes from diploid wheats and the comparative analysis with those from polyploid wheats. Genetika.

[B25] Van Herpen TWJM, Riley M, Sparks C, Jones HD, Gritsch C, Dekking EH, Hamer RJ, Bosch D, Salentijn EMJ, Smulders MJM, Shewry PR, Gilissen LJWJ (2008). Detailed analysis of the expression of an alpha-gliadin promoter and the deposition of alpha-gliadin protein during wheat grain development. Ann Bot.

[B26] Spaenij-Dekking EHA, Kooy-Winkelaar EMC, Nieuwenhuizen WF, Drijfhout JW, Koning F (2004). A novel and sensitive method for the detection of T cell stimulatory epitopes of α/β- and γ-gliadin. Gut.

[B27] Anderson RP, Degano P, Godkin AJ, Jewell DP, Hill AVS (2000). In vivo antigen challenge in celiac disease identifies a single transglutaminase-modified peptide as the dominant A-gliadin T-cell epitope. Nature Med.

[B28] Anderson RP, van Heel DA, Tye-Din JA, Jewell DP, Hill AVS (2006). Antagonists and non-toxic variants of the dominant wheat gliadin T cell epitope in celiac disease. Gut.

[B29] Sozinov AA, Poperelya FA (1980). Genetic classification of prolamines and its use for plant breeding. Ann Technol Agric.

[B30] Metakovsky EV, Akhmedov MG, Sozinov AA (1986). Genetic analysis of gliadin-encoding genes reveals gene clusters as well as single remote genes. Theor Appl Genet.

[B31] Metakovsky EV (1991). Gliadin allele identification in common wheat. II. Catalogue of gliadin alleles in common wheat. J Genet Breed.

[B32] Ruiz M, Rodriguez-Quijano M, Metakovsky EV, Vazquez JF, Carrillo JM (2002). Polymorphism, variation and genetic identity of Spanish common wheat germplasm based on gliadin alleles. Field Crops Res.

[B33] Aguiriano E, Ruiz M, Fité R, Carrillo JM (2006). Analysis of genetic variability in a sample of the durum wheat (*Triticum durum Desf*.) Spanish collection based on gliadin markers. Genet Res Crop Evol.

[B34] Kudryavtsev AM, Boggini G, Bnedettelli S, Illchevskii NN (1996). Gliadin polymorphism and genetic diversity of modern Italian durum wheat. J Genet Breed.

[B35] Alvarez JB, Moral A, Martín LM (2006). Polymorphism and genetic diversity for seed starage proteins in Spanish cultivated einkorn wheat (*Triticum monococcum *L. spp. *monococcum*). Genet Res Crop Evol.

[B36] Himi E, Noda K (2004). Isolation and location of three homoeologous dihydro-flavonol-4-reductase (DFR) genes of wheat and their tissue-dependent expression. J Exp Bot.

[B37] Mochida K, Yamazaki Y, Ogihara Y (2003). Discrimination of homoeologous gene expression in hexaploid wheat by SNP analysis of contigs grouped from a large number of expressed sequence tags. Mol Genet Genomics.

[B38] Nomura T, Ishihara A, Yanagita RC, Endo TR, Iwamura H (2005). Three genomes differentially contribute to the biosynthesis of benzoxazinones in hexaploid wheat. Proc Natl Acad Sci USA.

[B39] Shitsukawa N, Tahira C, Kassai K-I, Hirabayashi C, Shimizu T, Takumi S, Mochida K, Kawaura K, Ogihara Y, Muraia K (2007). Genetic and Epigenetic Alteration among Three Homoeologous Genes of a Class E MADS Box Gene in Hexaploid Wheat. Plant Cell.

[B40] Neve B, Froguel P, Corset L, Vaillant E, Vatin V, Boutin P (2002). Rapid SNP Allele Frequency Determination in Genomic DNA Pools by Pyrosequencing™. BioTechniques.

[B41] Wasson J, Skolnick G, Love-Gregory L, Permutt MA (2002). Assessing Allele Frequencies of Single Nucleotide Polymorphisms in DNA Pools by Pyrosequencing™ Technology. BioTechniques.

[B42] Schaart JG, Mehli L, Schouten HJ (2005). Quantification of allele-specific expression of a gene encodingstrawberry polygalacturonase-inhibiting protein (PGIP) using Pyrosequencing™. Plant J.

[B43] Somers DJ, Kirkpatrick R, Moniwa M, Walsh A (2003). Mining single-nucleotide polymorphisms from hexaploid wheat ESTs. Genome.

[B44] Konarev A, Gubareva N, Kornuchin D, Börner A (2005). Gliadin electrophoretic analysis of the genetic integrity of wheat (*Triticum aestivum *L.) accessions after frequent seed reproductions. Genet Res Crop Evol.

[B45] Eticha F, Belay G, Bekele E (2006). Species diversity in wheat landrace populations from two regions of Ethiopia. Genet Res Crop Evol.

[B46] Meyer M, Stenzel U, Hofreiter M (2008). Parallel tagged sequencing on the 454 platform. Nature Protocols.

[B47] Hutchison CA (2007). DNA sequencing: bench to bedside and beyond. Nucleic Acids Res.

[B48] Doyle JJ, Doyle Jl (1987). A rapid DNA isolation procedure for small quantities of fresh leaf tissue. Phytochem Bull.

